# Randomized controlled trial of probiotics for the prevention of spontaneous preterm delivery associated with intrauterine infection: study protocol

**DOI:** 10.1186/1742-4755-7-14

**Published:** 2010-06-30

**Authors:** Leticia Krauss-Silva, Maria Elizabeth L Moreira, Mariane B Alves, Maria R Rezende, Alcione Braga, Karla G Camacho, Maria Rosa R Batista, Clarisse Savastano, Antonio Almada-Horta, Fernando Guerra

**Affiliations:** 1 Health Technology Assessment Unit, National School of Public Health, Oswaldo Cruz Foundation, Brazilian Health Ministry, Brazil, R. Leopoldo Bulhões, 1480, Rio de Janeiro, 21041-210, Brazil; 2 Clinical Research Unit, Fernandes Figueira Institute, Oswaldo Cruz Foundation, Brazilian Health Ministry, Av. Rui Barbosa, 716, Rio de Janeiro, 22250-020, Brazil; 3 Institute of Mathematics, Federal University of Rio de Janeiro, Av. Athos da Silveira Ramos - 149, Rio de Janeiro, 21941-909, Brazil; 4 PROCEP, Pró-Cardíaco, R. General Polidoro, 142, Rio de Janeiro, 22280-003, Brazil; 5 Department of Obstetrics and Gynecology, Fernandes Figueira Institute, Oswaldo Cruz Foundation, Brazilian Health Ministry, Av. Rui Barbosa, 716, Rio de Janeiro, 22250-020, Brazil; 6 Federal University of Rio de Janeiro Medical School, Av. Brigadeiro Trompowski, Rio de Janeiro, 21044-020, Brazil

## Abstract

**Background:**

Spontaneous preterm deliveries that occur before the 34th week of gestation, and particularly before the 32nd week of gestation, have been strongly associated to intrauterine infection, ascending from vagina, and represent the largest portion of neonatal deaths and neurological problems. Bacterial vaginosis, characterized by a diminished or absent flora of lactobacilli and increased colonization of several anaerobic or facultative microorganisms, increases two times the risk of preterm delivery before the 34th week. Trials of antibiotics failed to show efficacy and effectiveness against spontaneous preterm birth related to bacterial vaginosis. Some studies indicate benefit from selected probiotics to treat genitourinary infections, including bacterial vaginosis.

**Objective:**

The purpose of this study is to evaluate the effectiveness of the early administration of selected probiotics to pregnant women with asymptomatic bacterial vaginosis/intermediate degree infection to reduce the occurrence of spontaneous preterm delivery and related neonatal mortality and morbidity.

**Methods/Design:**

Women attending public prenatal care services in Rio de Janeiro will be screened to select asymptomatic pregnant women, less than 20 weeks' gestation, with no indication of elective preterm delivery. Those with vaginal pH > = 4.5 and a Nugent score between 4 and 10 (intermediate degree infection or bacterial vaginosis) will be randomized to either the placebo or the intervention group, after written informed consent. Intervention consists in the use of probiotics, Lactobacillus rhamnosus GR-1 and Lactobacillus reuteri RC-14, 2 capsules a day, each capsule containing more than one million bacilli of each strain, for 6-12 weeks, up to the 24th-25th wk of gestation. Ancillary analyses include quantification of selected cervicovaginal cytokines and genotyping of selected polymorphisms. The randomization process is stratified for history of preterm delivery and blocked. Allocation concealment was designed as well as blinding of women, caregivers and outcome evaluators. The study will be supervised by an independent monitoring committee. Outcomes under study are preterm delivery (< 34- < 32 weeks of gestation) and associated neonatal complications: early neonatal sepsis, bronchopulmonary dysplasia, periventricular leukomalacia, necrotizing enterocolitis, and prematurity-related retinopathy; definitions were adapted from those recommended by the 2002 version of the Vermont-Oxford Network. Trial registration at NIH register: NCT00303082.

## 1- Introduction (Scientific background and explanation of rationale)

In the United States, where prematurity rates are higher than those in Western Europe, nearly 11% of babies are born premature and preterm delivery is associated to approximately 80% of fetal, neonatal and infants' deaths [1]. Studies in Brazilian urban areas indicate a prematurity rate above 10% - as high as the American rate [2,3].

About 25% to 30% of preterm deliveries occur due to medical indication and the remaining percentage occurs spontaneously [1-4]. Neonatal mortality and morbidity risks are inversely associated to gestational age at birth; spontaneous preterm deliveries that occur before the 35th week of gestation, and particularly before the 32nd week of gestation, have been strongly associated to intrauterine infection. [5,6].

Approximately half of the neurological disabilities found in children, including cerebral palsy, are attributed to prematurity; those disabilities limit the learning capabilities and social abilities during childhood and adult life and imply a significant overload for their families and for society. The subgroup of babies born before the 32nd week of gestation, which corresponds to around 2% of those born alive, represents the largest portion of deaths and neonatal neurological problems [5,6].

The cost of taking care of premature children in developed countries is very high [7]; in developing countries, like Brazil, the problem is composed by a low effectiveness of the assistance provided in nurseries, mainly that rendered in NICUs.

Important risk factors to spontaneous preterm delivery, such as age < 18 years, race, education, and bacterial vaginosis are associated with low social-economic level. The most important factor in spontaneous preterm delivery (SPTD), history of preterm delivery, increases 3 times the risk of a subsequent preterm delivery and is probably associated to underlying risk factors [4,8,9].

Among risk factors that could be modified, bacterial vaginosis (BV), an infectious condition, is the most important. Bacterial vaginosis is present in 15-20% of normal pregnant women in developed countries [8], and this proportion almost doubles in high-risk populations [10]. Studies conducted with pregnant and non-pregnant women suggest that BV in the lower genital tract is associated to infection of the upper tract by BV microorganisms; this could result in the inflammation of the decidua and chorio-amnion and cause subsequent preterm delivery as well as fetal and neonatal infection [5,6].

BV increases two times the risk of preterm delivery before 35th weeks' gestation [8]. Prematurity thus originated increases the probability of certain complications, such as early sepsis, bronchopulmonary dysplasia, periventricular leukomalacia and necrotizing enterocolitis [11-14], which may cause the referred long term disabilities as well as death.

Bacterial vaginosis (BV) is a modification of the vaginal flora characterized by a diminished or absent flora of lactobacilli, which increases the vaginal pH, and a significantly increased colonization of several anaerobic or facultative microorganisms, mainly Gardenerella vaginalis, Prevotella sp, Bacteroides sp, Mobiluncus sp, gram positive cocci, and genital mycoplasma (Mycoplasma hominis and Ureaplasma urealyticum). Bacterial vaginosis is a condition in which there are no parabasal cells and leukocytes are rare, in a similar way to what occurs in women whose mucosa is normal [15].

Although BV is the most prevalent vaginal disorder in adult women in the world, its pathogenesis is still not clear. In most cases, the condition is asymptomatic; the vaginal secretion presents scarce inflammatory signs, with very few leukocytes. That is why the term used to define the condition is "vaginosis" and not "vaginitis" [15]. The reason why most women who have BV do not present inflammatory signs, even in the presence of abnormal flora, is unknown yet. To understand the response of vaginal immunity to microbial invasion may be crucial to avoid adverse results associated to bacterial vaginosis (BV).

The Nugent method is a standardized method, relatively simple and inexpensive, designed to evaluate bacterial vaginosis. It requires Gram-staining the vaginal smear and was developed to obtain an objective and reproducible laboratorial test, thus differing from the traditional Amsel method, which includes clinical criteria. The Nugent score classifies the vaginal flora according to the semi-qualitative presence of lactobacilli, Gardenerella vaginalis and bacteria similar to Gardenerella (such as Prevotella, Bacteroides and Porphyromonas sp), and Mobiluncus sp. It is highly associated to BV clinical signs and vaginal pH [16,17]. It also provides a gradation of disturbances of the vaginal flora: a Nugent score from 0 to 3 indicates a normal flora; from 4 to 6 indicates an intermediate flora and from 7 to 10 indicates BV [16].

The relatively low sensitivity of BV tests for premature birth, close to 30% (both Amsel's and Nugent's), is similar to the performance of other screening tests for spontaneous premature birth in asymptomatic women, such as the Bishop test, cervical ultrasound and fetal fibronectin [18]. Their sensitivity is relatively low according to some cohort studies and large case-control studies; an improved performance can be obtained in populations at higher risks [8-19].

It is important to note that the so-called intermediate level (Nugent score 4-6) presents risk for premature birth close to that of BV (score 7-10) [20,21]. The inclusion of the intermediate degree apparently increases significantly the sensitivity of the method, without extra costs, while providing an earlier detection of the vaginal disturbance.

Elevated vaginal pH and neutrophils are strongly associated to early spontaneous preterm birth [22]. On the other hand, data published by a large study showed that the use of the vaginal pH < 4.5 as a cutoff point to preliminarily sort pregnant women, between the 8th and the 22nd weeks, brings about the exclusion of around 40% of asymptomatic women and one third of the spontaneous preterm births less than 32 and also less than 35 weeks, although almost 50% of the remaining (non excluded population, 60%) is classified between 0-6 [21].

Assuming that: a) the sensitivity of the Nugent test is around 50% (BV + Intermediate stage), b) the change of the vaginal ecosystem that results in BV directly affects the vaginal pH (reduction of lactobacilli), one of its classical markers, and c) the determination of the vaginal pH is simple, immediate and relatively cheap, the proposal of using the vaginal pH as a pre-screening step of the Nugent test seems justified as a way to increase the efficiency of the whole screening process.

The association between infections of the upper urinary tract with preterm delivery (premature birth) in BV asymptomatic women supported the trials on antibiotics to prevent preterm delivery in these women. But the results of these trials were contradictory [23-25]. To resolve these contradictions, NIH/DHSS (USA) produced a well designed large trial on the subject. Nevertheless, its results did not show any trend whatsoever of benefit towards antibiotic-therapy (metronidazole associated to erythromycin). Besides, the study showed negative results in the subgroup of patients with history of preterm delivery [26]. Those results could be attributed, in part, to bacterial resistance, to the timing of the intervention (possibly too late), to the toxicity for the fetus; it could also be partially due to a particular form of inflammatory process apparently associated to BV [27-29].

Thus, when examining interventions to prevent preterm delivery associated to intrauterine infection, originated from bacterial vaginosis, it is important to consider an alternative that might overcome the limitations of antibiotic-therapy, as mentioned above. The so-called probiotics, a kind of bacteriotherapy, has been studied lately and is the object of the present study.

Probiotics have been tested for treating infectious, inflammatory and allergic conditions, mainly those that occur in the intestinal, genitourinary and respiratory tracts [30,31]. One of the important preliminary steps of a trial of probiotics is the previous design/selection of the bacteria (probiotics) to be tested, based in requirements deemed as essential [32].

Some studies indicate that selected probiotics are efficacious against moderate urogenital tract infections, including BV [33-38]. Nevertheless, there is no evidence of the effectiveness of probiotics for BV related conditions like preterm birth and neonatal morbidity.

### Invasive mechanism and causative role of intrauterine infection

Microorganisms may reach the amniotic cavity and the fetus, generally, by ascending from the vagina and bottom of the uterus, according to hystological and microbiological studies. Some bacteriae are more frequently isolated from the amniotic cavity of women in preterm labor and whose membranes are intact: Ureaplama urealyticum, Fusobacterium spp and Mycoplasma hominis. Other organisms associated to bacterial vaginosis, such as Streptococcus agalactiae, Gardenerella vaginalis and Bacterioides spp, were also found. So far, there is no outstanding evidence that viruses might be linked to the genesis of preterm delivery [5,6].

Studies on biological markers of intrauterine and intra-amniotic infection such as FNF and interleukin-6, are consistent with histological and microbiological observations: it takes an average of 7 or 8 weeks for a preterm delivery, sepsis or fetal/neonatal death to occur, after a positive test. Such evidence as a whole suggests that the sub-acute inflammatory process is associated with spontaneous abortion and preterm delivery [5].

Therefore, existing evidence supports a role of intrauterine infections in significant causes of spontaneous premature birth and fetal and neonatal infections, thus fulfilling the requirements of biological plausibility, temporal relation, consistency and force of association, gradient dosage-response and specificity.

Intrauterine infection may not result however in premature birth and often it does not; this would indicate that the fetus or the pregnant woman has dealt with the infection in order to continue the pregnancy, regardless of the fact that the infectious process has not been cured. Most patients presenting with asymptomatic BV go through a regression process that takes several weeks, to normal vaginal conditions [10].

The general consensus on the host and microbial interactions in the genital tract is that systemic immunity does not perform a dominant role, but the immunoregulatory mechanisms of the local response do. The vagina is different, to a certain degree, from other human mucosal surfaces because this niche must be tolerant with xenobiotic molecules (gestation) [15].

Simhan et al [27] proposed a model to explain the link between inflammatory response and modifications of cervicovaginal ecosystem and the clinical picture. In this model, the existence of individualized patterns of immunological response leads to different types of evolution, that is: when pregnant women are hypo-respondent, the immune response would not be able to control bacterial infection and patients would be predisposed to ascending intrauterine infections. In the opposite case, of a hyper response, development of an exaggerated inflammatory response would occur, with symptoms of vaginitis which could result in preterm births. The ideal response would be the development of an inflammatory response proportional to the severity of the aggression.

An immunomodulatory role has been proposed for IL-10 along with a call for the examination of not only pro-inflammatory but also anti-inflammatory cytokines and associated genetic polymorphisms to differentiate between hyper- and hyporesponders [39].

### Hypo-response

Cauci et al [15,40,41] observed that the innate response of the vaginal mucosa of women with BV, which should result in the proliferation of neutrophils, generally does not occur. Epithelial cells produce cytokine IL 1B but the production of IL8 (induced by IL 1B), that, on its turn, induces production of neutrophils, is prevented by hydrolitic enzymes and by proteolytic enzymes (sialidases and prolidases) produced by the BV bacteria. Cauci et al [40-42] also observed that the presence of those enzymes in the vaginal fluid, accompanied by (in interaction with) an elevated pH (that fosters the activity of these enzymes) increases more than ten times the risk of premature birth.

Moreover, Simhan et al [27] observed that pregnant women with low levels of IL-1B, IL6 and IL 8 cytokines in the cervical fluid, obtained between the 8th the 20th week, are more susceptible to developing clinical choroamnionitis.

Regarding the immunospecific response, Cauci et AL [43] found out that part of the women that have BV do not produce a specific answer to BV agents. One of the reasons for this hypo-answer is the extensive degradation of Ig A and Ig Ms developed by the sialidases produced by BV bacteria, high levels of sialidases appears to be associated with resistance to antibiotic therapy for BV or with early reinfection [41,44].

### Hyper-response

Several studies investigated the role of hyper-responsiviness in the genesis of spontaneous preterm delivery. They observed that the increase of cytokines, typically related to inflammatory processes in the mucosa of the cervicovaginal area, is linked to invasion of the lower and upper gynecological tract by atypical bacteria, and is associated to preterm delivery [24,27,45-47].

The increase of those cytokines has also been associated to serious neonatal damages: pneumonia, sepsis, necrotizing enterocolitis, periventricular leukomalacia, periventricular bleeding, cerebral palsy and bronchopulmonary dysplasia [14,48,49]. The study conducted by Genc et al [50] showed association of high concentration of IL-1B with preterm birth. It also found an even higher association of a low ratio IL-1ra (natural antagonist of the IL 1B)/IL 1B, that is, a high concentration of "free" IL-1B, with preterm delivery. It also observed a high sensitivity (78%) of the IL ratio - 1ra/IL-1B - in predicting preterm delivery.

Those findings are being complemented by studies about genes involved in the inflammatory reactions related to the ascending vaginal infections that precede preterm delivery. A study conducted by Macones et al [51], in the USA, found high association of the rare allele of the TNF gene (TNF2) - that modulates inflammatory processes towards hyper-response - with preterm delivery, and a powerful multiplicative interaction (OR = 6; p = 0.007) of that allele with bacterial vaginosis (symptomatic). Moreover, Simhan et al [52,53] found that a polymorphism of TNF (-308) is associated with chorioamnionitis, and a polymorphism of the promoter of interleukin 6 (-174) is associated with spontaneous preterm delivery; black pregnant women lacked the protective allele related to IL-6. A similar but more comprehensive study was conducted with Australian women [54].

The above studies suggest that it might not be appropriate to exclude but to treat and then include pregnant women with vaginal symptoms of infection in trials of interventions to avoid preterm delivery [28]. Maternal and fetal polymorphisms associated to hypo- response have not been clearly identified.

### Probiotics: basic concepts

Some studies have demonstrated that the microbiological environment may overcome the marked virulence of a given bacterial strain in causing the disease, indicating that the importance of environmental factors is intertwined with the issue of microbial replication. The exogenous or endogenous bacteria that carry pathogenic pre-requirements must have replication dominance so that the disease occurs. The acknowledgement that a microbial species can inhibit a different type of microbe has provoked interest in exploring this phenomenon for the wellness of human beings [55]. By definition, probiotic bacteria are live microbes and have beneficial effects on human health [30,56].

The correction of properties of a non balanced indigenous microbiota - in this case, manipulation of vaginal microbiota in order to interrupt the infectious/inflammatory ('allergic') process that leads to preterm delivery - constitutes the rationale of probiotic therapy. Some studies indicate that a transient colonization may be important and sufficient since the non-balanced microbiota can be corrected by a short probiotic intervention [30] and this may be relevant in the case of BV during pregnancy.

Due to the poly-microbial nature of BV, cure, treatment and control of recurrence are more complex processes than those related to diseases caused by a single infectious agent [57].

The evidence that lactobacilli protect against vaginal infections because they produce lactic acid and therefore maintain a pH higher than 4.0-4-5 have been established long ago. Furthermore, lactobacilli inhibit the growth of a large number of opportunist vaginal microorganisms, including E.coli, Candida albicans, G.vaginalis, and Mobiluncus sp, because they produce hydrogen peroxide (H2O2), which further decreases vaginal pH, as well as other inhibiting metabolites [42,57].

Moreover, many of the probiotic effects are not related to changes in microbiota only, as indicated by cultures. Actually, an important part of the beneficial effects of probiotics is related to its immunomodulating effects: increase in immunologic activity as well as of anti-inflammatory activity [30].

Studies on the intestinal microbiota, for example, indicate that it provides immunomodulation of the inflammatory activity, thus avoiding aberrations that might lead to disease. A healthy homeostasis might be attained by optimizing the balance between pro and anti-inflammatory cytokines. The effects of tolerance (toleragenic) of the intestinal flora may be mediated by the production of regulatory T cells (T and T helper), that characterize the intestinal immunological system and induce suppressing cytokines that fight hypersensitivity and inflammation [30].

However, uncertainties about the mechanisms involved in vaginal immunity and on the set of metabolic characteristics of the bacteria to be used as probiotics, add difficulties to design effective and safe interventions, related not only to BV, but also to the outcomes preterm delivery and neonatal morbidity [57].

### Design/development of the alternative probiotics: criteria and studies related to the biological plausibility that probiotics may specifically cure the condition Bacterial Vaginosis and may prevent spontaneous preterm birth

Some studies focus on attributes considered biologically desirable for probiotics to treat the BV condition. These attributes, which may turn a probiotic effective to treat BV, have been tested in vitro and in vivo, using some specific probiotics, as detailed ahead.

Women whose vagina is colonized by lactobacilli that produce H2O2 have less possibility of developing vaginosis. The maintenance of vaginal pH close to 4.0, through the production of lactic acid and H2O2, is essential for vaginal health. The production of H2O2 is thus considered an important characteristic of a lactobacilli strain intended to become a probiotic in the BV condition. Studies in vivo demonstrated adhesive potential to the urogenital epithelium of some strains [36,37]; an in vitro study showed greater or lesser adhesion to vaginal epithelium for as little as 60 species studied [59]. When lactobacilli are introduced orally, they may move from the rectum to the vagina within one week [35]. Studies in vitro, using molecular typing, observed the permanence of exogenous lactobacilli for several weeks after being introduced [57,58].

The ability of probiotic lactobacilli strains to induce apoptosis of bacteriae they are competing against to get nutrients (growth antagonism) and for vaginal niche colonization (adherence to vaginal epithelial cells and multiplication), as well as to escape the immune response of the host, added to/through their potential of producing H2O2, must be considered as parameters to select strains. Not all lactobacilli strains, producers of H2O2, are capable of effectively colonizing vaginal niches [57]. The exclusion by apoptosis of BV bacteria was shown in in vivo studies for some lactobacilli strains [57,58], as well as the in vitro and and in vivo growth inhibition of BV bacteriae, in a more or less intense way [57-59], the production of H2O2 in vitro and in vivo [36,59]. Reid considers the possibility that the apoptosis/displacement of bacteriae may also happen through immunomodulation [33].

Other attributes, characteristics and additional metabolic mechanisms, often associated to the previously mentioned abilities, are also relevant to increase the chance of a probiotic changing into normal the vaginal microecology of a flora associated to bacterial vaginosis. The production of bacteriocins that inhibit the synthesis of enzymes by BV bacteria, the already mentioned sialidades and prolidases - that are markers of virulence -is one of the important generic attributes of these probiotics.

For example, an important metabolic characteristic of a probiotic is its ability to inhibit excessive production of poliamines by BV bacteriae, that is, to present increased activity of the enzyme arginine deiminase. When this enzyme (bacteriocin) deprives the environment of arginine, it propitiates the colonization of lactobacilli - fostering the apopotosis of anaerobic bacteriae -, it inhibits the growth of these bacterie and the consequent inflammatory activity [57]. The clinical recovery of patients with BV, after the intake of a specifc lactobacilli strain with increased activity of the enzyme arginine deiminase, was observed along with a progressive decrease in the concentration of poliamines in the vaginal secretion [57].

The importance of the degree to which the referred attributes are present for the efficacy of a probiotic is still not clear [58,59]. In general, the studied lactobacilli strains are not homogeneous regarding those attributes. The production of H2O2 and that of the specific bacteriocin arginine deiminase, for example, are mutually exclusive. Such observations by different studies have resulted in the consensus that a probiotic must have more than one strain of lactobacilli that are complementary as to requirements considered fundamental [36,57,59].

Another research group that focuses on the question of hyper-response suggests that lactobacilli must show anti-TH2 activity in order to counterpoise/avoid "allergic" answers. Pochard et al [60] observed anti-TH2 activity in lactobacilli strains tested in vitro, which inhibited the production of cytokines that characterize the allergic reaction of the respiratory tract (IL4 e IL5). This ability of lactobacilli has not been tested yet regarding the BV condition.

#### Safety of probiotics

Besides the already mentioned desirable attributes of an anti BV probiotic - adherence to the epithelial cells of the vagina, apoptosis of pathogenic microbes, production of H2O2 and of bacteriocins antagonistic to the growth of atypical microorganisms, as well as anti-TH2 activity - a probiotic must be safe.

Lactobacilli and bifidobacteriae are, in principle, considered to be safe because they are components of the comensal human flora and due to the fact that they have been used for a long time in food industry and in douches, even by pregnant women, without harmful effects [31,56,58].

Although lactobacilli have been identified in blood cultures and cultures of other organs, infections by lactobacilli are rare (30). Positive hemocultures for lactobacilli occurred in 0.24% of patients in a Finish study and in 0.1% in French patients; the infections occurred in patients with immunodeficiency [31,32]. The induction of infection has already been directly or indirectly tested by several studies on probiotics and none of the strains caused morbidity [58].

### Evidence found by trials on the effectiveness and safety of tested lactobacilli against BV

The choice of a probiotic, like that of any medical technology, requires that its effectiveness and safety are verified and so, the evaluation of these attributes, conducted by clinical trials _ preferably those randomized, controlled and double-blind_ is an important requirement for its regular use in human beings.

### Summary of trials available on the subject

a. Reid et al, 2001 [35] - Prospective study with a group of 10 asymptomatic women, with history of recurrent urogenital infection, including 6 asymptomatic cases of intermediate degree infection/BV, treated with an oral solution containing selected lactobacilli (Lactobacillus rhamnosus GR-1 e Lactobacillus fermentum TC-14), twice a day, for 14 days. No adverse side effects were reported and adherence to treatment was considered excellent. The 6 asymptomatic cases of intermediate degree infection/BV were cured after the first week of treatment and remained normal during follow-up (between 1 and 3 months).

b. Reid et al, 2001b [37] - Randomized controlled trial, comparing 4 probiotics: 3 of them were formed of 2 selected lactobacilli strains (Lactobacillus rhamnosus GR-1 and Lactobacillus fermentum TC-14) in different dosages: 1 dosage (2 groups), 2 dosages (1 group); the 4th group was considered the placebo because the lactobacilli used had no outstanding properties. Sixty percent (60%) of women presented BV or intermediate degree infection; 1/3 of them presented BV at the beginning of the study. Forty-two (42) asymptomatic women were randomized; the technician that evaluated the Nugent was blinded, as well as the researchers and the patients, except those receiving 2 daily dosages. Probiotics were orally taken (capsules) for 28 days. No adverse side effects were reported and adherence to treatment was considered excellent. The results were favorable to probiotics as compared to placebo; the best results against BV were observed in the group of patients who received 2 dosages a day (close to 90% effectiveness for BV). These results remained unchanged for a month after the treatment had finished.

c. Reid et al, 2003 [34] - Randomized, double-blind placebo-controlled trial (patients and researchers) to test the safety and prevention/treatment of asymptomatic urogenital infections, including BV. A population of 64 asymptomatic women in their fertile years used, once a day, a capsule with 2 types of selected bacilli (Lactobacillus rhamnosus GR-1 and Lactobacillus fermentum TC-14), with a concentration of each bacillus per capsule superior to 1 million or of placebo, for 60 days. The adherence of patients was considered excellent. Patients, monitored throughout the study, did not report side effects. Close to 40% of the cases of asymptomatic BV improved (they became intermediate stage) at the end of treatment, in comparison with 13% of the placebo group (p = 0.02). There was also significant reduction in the presence of Monilia and of E.coli in the group treated, versus the placebo group. However, the efficacy decreased after a 30 day-follow up.

d. Shalev, 2002 [38] - Randomized controlled trial, with crossover, comparative of the ingestion of yogurt containing lactobacilli acidophilus and pasteurized yogurt, in a total of 46 women for prophylaxis of recurrent BV. Significant statistical increase was observed as to the number of lactobacilli acidophilus in the vaginal mucosa and also a significant statistical decrease of BV cases (p < 0.001) occurred during the period of treatment with lactobacilli, as compared to the period when groups ingested pasteurized yogurt.

The above studies _small but consistently in favor of lactobacilli probiotics _ indicate that the ecologic treatment of BV through the colonization of vagina by exogenous lactobacilli is an alternative worth trying, both for BV and preterm delivery. However, the effectiveness of probiotics regarding the consequences of BV, i.e., preterm delivery and neonatal morbidiy, although plausible, has not been reported yet. It is important to remark that, despite the fact that none of the mentioned trials enrolled pregnant women, Lactobacilli rhamnosus have been used in a controlled clinical trial in pregnant women [61], side effects not being observed.

On the other hand, the probability of a test yielding benefits for health depends on the frequency of the problem, the ability of the test to detect the condition and to what extent early treatment is better than late intervention [62]. The lead time (time lapse between the detection and the moment in which the condition to be avoided might have occurred without the screening) needed to change favorably the natural history of a problem, varies in different diseases, different cases and depends on the treatment [63].

In this project, it is assumed that the necessary lead time to prevent spontaneous preterm delivery, associated to infection, depends on the moment (gestational age) at which vaginal infection occurs, on the kind of infection and on the mother (fetus)'s response to it, as well as on the moment the test is conducted regarding the development of the ominous infectious/allergic process and on the ability of the treatment to subdue the infection at the occasion (timing) it is conducted.

Thus, the rationale for the trial is based on the hypotheses that: a) the observed lack of efficacy of antibiotics may, in part, be attributed to lead time - the intervention would have occurred too late _, in part to bacterial resistance, in part to toxicity of the antibiotics used and, finally, in part, to an inflammatory process similar to allergy [27-29], and b) probiotics have more favorable attributes (bacteriotherapy plus immunomodulation) and, supposedly, less adverse events, to deal with bacterial vaginosis and preterm delivery, provided that they are given within an adequate lead time and dosage for an effective intervention.

## 2. General Objectives

To estimate the effectiveness of the early administration of specially formulated probiotics to pregnant women with bacterial vaginosis or an intermediate degree infection to prevent the occurrence of spontaneous premature birth and related neonatal mortality and morbidity.

### 2.1. Specific objectives

a- To assess the presence of bacterial vaginosis and intermediate degree infection in asymptomatic pregnant women, with no risk/indication of elective preterm delivery, admitted to prenatal care after the 8th and before the 20th week of gestation.

b- To investigate whether the study intervention can lower the vaginal pH and the Nugent score.

c- To asses the effectiveness of an early intervention with special probiotics to treat bacterial vaginosis/intermediate infection and to prevent spontaneous preterm delivery and associated neonatal/infant conditions in positive women, according to item *a*, by conducting a controlled, randomized, double-blind trial.

d- To analyze the association between the concentration of selected cytokines at cervicovaginal mucosa and the Nugent score, and the < 34 weeks' gestation prematurity rate, in both study subgroups.

e- To analyze the association of selected genetic polymorphisms and the occurrence of a) bacterial vaginosis or intermediate degree infection and b) preterm birth, in both study subgroups.

## 3. Methods

Asymptomatic pregnant women, admitted after the 8th and before the 20th week of pregnancy in selected public prenatal care services, in the city of Rio de Janeiro, will be evaluated to identify a) excluding clinical conditions, mainly those related to the presence of pathological conditions associated with elective preterm delivery, to symptomatic vaginal conditions and to current, or within the past two weeks, use of antibiotic therapy and b) pregnant women with previous history of preterm delivery. Gestational age will be determined by the date of the last menstrual period and through ultrasound results, the last one being privileged. Women who test positive to syphilis, toxoplasmosis, gonorrhea and HIV will be excluded. Other absolute exclusion criteria are: multiple gestation, istmo-cervical incompetence (cerclage in current gestation), fetus with major congenital malformations in current gestation, insulin dependent diabetes mellitus, systemic arterial hypertension under medication, antibiotic therapy after the 8^th ^week of the current gestation, clinical suspicion of low urinary tract infection, chronic asthma requesting intermittent therapy, continuous or recent corticotherapy (up to one month), hemolytic perinatal disease; systemic eritematous lupus. Relative exclusion criteria, i.e, only if observed at the moment of the evaluation, are: bacterial vaginosis or tricomoniasis with laboratory confirmation, HPV (those with macroscopic lesions in the genitalia or microscopic pre-cancerous lesions).

After written informed consent, vaginal pH assessment will be performed; patients with a pH < 4.5 will be excluded. Then, cervicovaginal smear will be obtained to evaluate the presence of BV or intermediate degree infection, using the Nugent method. Women with a Nugent score under 4 will be excluded from the trial.

### Ancillary analyses

Vaginal fluid and oral mucosa samples will also be obtained to allow for the evaluation of selected cytokines (IL-1beta, IL-1ra, IL-6, IL-8 and IL-10) and related genetic polymorphisms, respectively, from women with a pH> 4.4 who have consented. Furthermore, vaginal smear, cervicovaginal fluid and oral samples will be collected from a subsample of the women with a pH < 4.5 for comparison purposes. Gram-stained vaginal smears will be further analysed to determine the number of leukocytes per epithelial cell and the proportion of parabasal epitheliocytes to allow for the detection of moderate or severe aerobic vaginitis.

### 3.1. Randomization process

It was stratified for the factor history of preterm delivery (HPD), and blocked by using a permuted block design. Stratified randomization was attained by producing one blocked and randomized list for each stratum. Blocks of size 8 were specified for the strata of non HPD; for the group of HPD, blocks of size 4 have been adopted. The sequence for randomization was electronically generated by an independent research assistant.

Women with a Nugent score between 4 and 10 will be randomized, after a second written informed consent, as soon as possible. Research nurses responsible for allocation are not directly involved with prenatal care, i.e., with clinical screening. Allocation will be concealed by sealing both placebo and intervention capsules, identical in aspect, in sequentially numbered identical containers according to the allocation sequence. Besides the pregnant women, doctors/nurses and researchers/evaluators (assessment of outcomes, including Nugent score and other lab procedures) are to be blinded to the randomization process.

The trial will test two lactobacilli strains, Lactobacillus rhamnosus GR-1 and Lactobacillus fermentum RC-14, the latter presently denominated Lactobacillus reuteri RC-14, developed and studied by Reid et col [33-36] as earlier mentioned. Each capsule contains an amount superior to one million bacilli of each strain. Each participant will receive, from the research nurse, one box with six bottles, box and bottles equally numbered, of probiotics/placebo, each bottle containing 30 capsules, corresponding to a maximum intervention period of 12 weeks. Participants will be then instructed to maintain the box at the refrigerator (not at the freezer) and to take two capsules per day, along a period that may vary from six to twelve weeks, depending on the participant's gestational age at the moment of enrollment in the study. Enrolled women will be free to contact the research assistant nurses or the research doctor; one week after randomization, they will be contacted by telephone for information about treatment. More detailed questioning will be made at each routine prenatal visit until the completion of the trial (around the 24-25th week of gestation) regarding adhesion/adherence, adverse events and clinical intercorrences, according to a previously designed form. Adherence will be fostered, when appropriate. At the post treatment prenatal care visit, randomized women will be asked to bring back all bottles; capsules remaining in the bottles they return will be counted. Adherence is defined as at least 6 wks of treatment.

At the post-treatment prenatal care visit, vaginal pH will be assessed; cervicovaginal smear and fluid will be collected to determine the post intervention Nugent score and levels of the selected cytokines in order to evaluate changes in those parameters (secondary results).

A detailed and extensive protocol was developed. Standard Operational Procedures were elaborated to cover the logistics and operational aspects of the trial. They cover not only details related to the whole screening process and materials' collection, transportation and storage but also elements that are relevant to the randomization process, including creation and protection of the allocation sequence, labeling of bottles and boxes, as well as the filling in and sending out the Case Record Forms.

A special software has been developed to acquire and store collected information. A workshop for training purpose and a pilot study were carried out in each of the services involved in the study; the protocol was revised accordingly.

**Figure 1:**
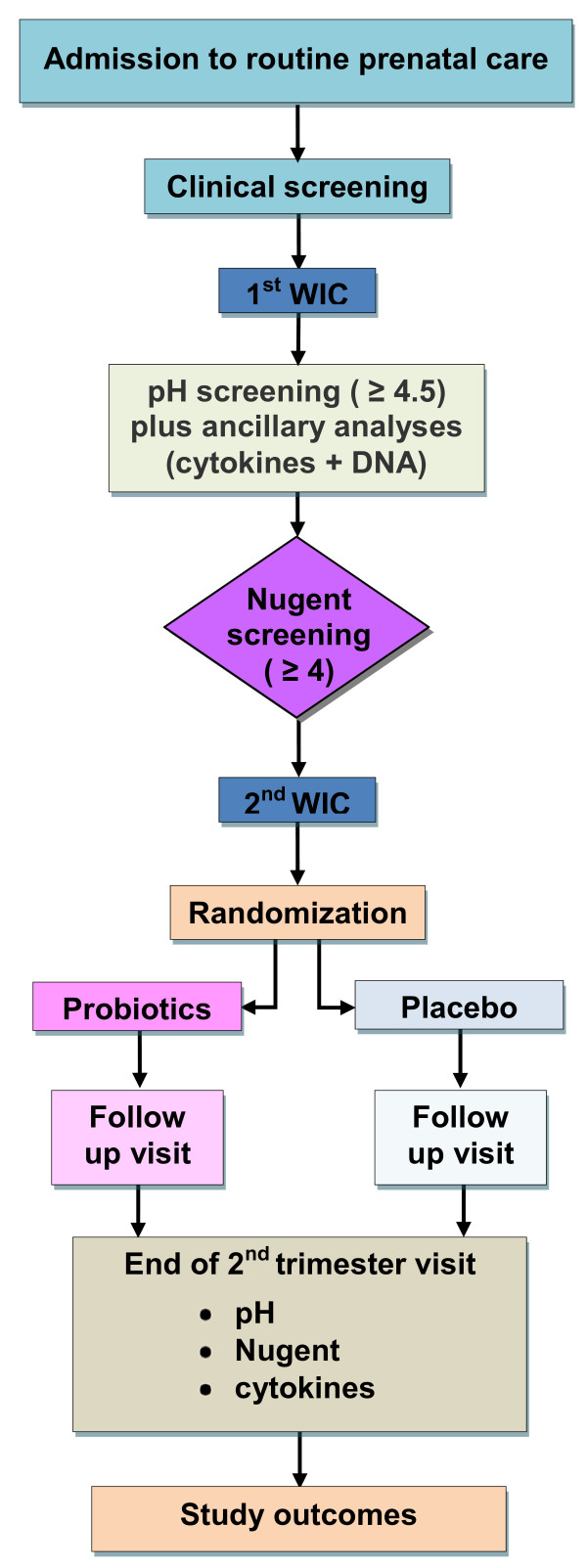
**Flow diagram according to the trial protocol**.

Patients flow in accordance to the protocol is summarized in Figure 1.

The outcomes to be studied will be preterm delivery < 34 and < 32 weeks of gestation and associated morbidity and mortality: early neonatal sepsis, bronchopulmonary dysplasia, periventricular leukomalacia, necrotizing enterocolitis, and prematurity-related retinopathy. Definitions of these conditions are similar to those used by the Vermont-Oxford Network, published in 2002.

A more detailed account of selected procedures and definitions is presented below.

### 3.2. Laboratory Methods

#### Measurement of vaginal pH

Vaginal pH will be measured by a strip with discrimination of 0.5, to evaluate women with pH higher than or equal to 4.5 and higher than or equal to 5.

#### Collection of clinical specimen for bacteriological analysis

Nugent Method - A swab will be used to obtain a cervicovaginal smear that will be Gram stained to be examined and interpreted according to the criteria described by Nugent, Krohn and Hillier [16]. A complementary analysis of the vaginal smear will be performed (400 × magnification, phase contrast microscope): the determination of the number of leukocytes per epithelial cell and the proportion of parabasal epitheliocytes to allow for the detection of moderate and severe aerobic vaginitis [64].

#### Quantitative tests of cervicovaginal cytokines

Cervicovaginal fluid will be collected by using 2 Dracon swabs (Fisherland, Fisher Scientific, Pittsburgh, PA, USA). Each swab will be placed in the vagina and cervical os for 10 seconds, thus reaching saturation. Next, each of them will be placed in a polypropylene plastic tube containing tampon-saline phosphate (PBS). The material will be stored at -20C. The quantification of the selected inflammatory cytokines (IL-1beta, IL-1ra, IL-6, IL-8 and IL-10) will be conducted by an immunoenzymatic test of capture or "sandwich-type" test, using R & D Systems, or, if possible, a special LUMINEX platform in order to broaden the spectrum of cytokines to be analyzed.

#### Genetic polymorphisms detection

A swab of the oral mucosa will be obtained and the DNA will be extracted by the conventional procedure [64]. Relevant single nucleotide polymorphisms will be investigated for TNF, IL-1beta, IL-1ra, IL-6 e IL-10. They will be genotyped with the PCR and sequence specific primers (PCR-SSP).

### 3. 3. Criteria to Diagnose Neonatal Morbidity

The criteria used to identify study morbidty were adapted from those defined by the Vermont Oxford Network in 2002.

- Early neonatal sepsis (72 h) - blood for the leukogram should be obtained during the first 72 hours; diagnosis will be reached by analyzing the neutrophilic index (ratio between immature neutrophils/total neutrophils). Diagnosis will also be done in case of positive hemoculture.

- Periventricular leukomalacia -evaluation will be conducted in newborns less than 32 weeks' gestation by performing a transfontanel ultrasound. To be considered cystic periventricular leukomalacia, multiple small periventricular cysts *must *be identified.

- Bronchopulmonary dysplasia - evaluation should be conducted in newborns less than 32 weeks' gestation, using the criteria of a) suggestive X-Ray and b) newborn need of any supplemental oxygen on the date of week 36, gestational age.

- Necrotizing enterocolitis - evaluation should be conducted in newborns with less than 34 weeks, using both clinical and radiographic criteria.

- Newborn retinopathy - evaluation will be conducted by a neonatologist trained in performing indirect ophthalmoscopy in newborns. The exam will be realized at 4 weeks after birth in newborns less than 1500 g.

### 3.4. Sample size

The estimation of the sample was based on the following information:

a) international and national data (very scarce) on the incidence of, and microorganisms associated with bacterial vaginosis and intermediate degree infection in pregnant women, taking into consideration different concepts and degrees of severity of those conditions and also, different populations and techniques used to identify and evaluate it [21,23,24] as well as the probabilities of natural recovery from vaginosis [10];

b) an estimate of the intersection of intermediate degree/BV scores and pH cutt off point (4.4), i.e., the percentage of pH positive (> 4.4) asymptomatic pregnant women who present a normal (0-3) Nugent score);

c) an estimate of the percentage of spontaneous preterm delivery expected in the placebo group [21,25];

d) an estimate of the effectiveness of probiotics to be tested in the study.

An initial loss, close to 20% of the pregnant women admitted for routine prenatal care, was supposed to occur due to the clinical exclusion criteria. The use of the vaginal pH < 4.5 parameter as a cutoff point to sort the pregnant women in whom the Nugent test will be performed, would bring about the exclusion of around 40% of the pH tested women; it also would imply the loss of one third of the less than 34 week spontaneous preterm deliveries expected to occur in the population that passed the clinical screen, although around 45% of Nugent score of the remaining population (after clinical and pH screens) would fall between 0-6 [25].

On the other hand, for that remaining population, the estimated incidence of BV plus intermediate degree would be 85%, which would result in the exclusion of around 15% of the population that passed the clinical and pH screens [25]. Therefore, the combination of the pH and Nugent criteria was expected to exclude near 50% of the pregnant women who passed the clinical screen; it also would imply the loss of about 20% of the less than 34 week spontaneous preterm deliveries expected to occur in the population that passed the clinical, pH and Nugent screens. The rate of spontaneous preterm deliveries < 34 weeks was estimated to be 6% in the placebo group and close to 3% in the intervention group, i.e., efficacy was supposed to be 50%; for spontaneous preterm deliveries under 32 weeks, the corresponding estimates would be close to half of those percentages.

Based on a significance level of 0.05 and a 80% power to show differences in rates of less than 34 week spontaneous preterm deliveries, the population to be randomized was estimated to be 1500 pregnant women (to show 50% efficacy regarding spontaneous preterm deliveries less than 34 weeks gestation). Taking into consideration the previous losses plus 5% refusals to give written informed consents as well as 15% loss related to adherence to treatment, a sequence of nearly 3500 pregnant women shall be screened in order to obtain the desired sample.

### 3.4. Data analysis

A post randomization table will comprehend the distribution of all relevant variables. Outcomes will be analyzed according to intention to treat and effective treatment. The Student t test, the X2 test for proportions and the Fisher test will be used in the preliminary data analyses. In those cases where the Student test does not apply due to non Gaussian distribution, the Mann-Whitney test will be used. Outcomes will also be analyzed according to subgroups of selected risk factors at admission and categories of severity of the infectious process, according to the study biological markers. The association between knowledge based selected factors and dichotomous or continuous outputs will be investigated under the framework of generalized additive models to handle non linear predictors as well as non Gaussian responses.

### 3.5. Ethical issues

The trial was approved by an Institutional Review Board and by the National Review Board (CONEP). The trial will be monitored by an independent data monitoring committee.

The trial conduction will follow ICH/GCP Regulation and the local regulation for clinical trials.

### 3.6. Registration

The study has been registered at the NIH register platform; its identifier is NCT00303082. The study has recently finished recruiting patients.

Sponsors: The trial received grants from FIOCRUZ/Brazilian Health Ministry, SAS/Brazilian Health Ministry and Rio de Janeiro State Research Foundation.

## Competing interests

The authors declare that they have no competing interests.
